# Laparoscopic Cholecystectomy in Acute Calculous Cholecystitis: A Secondary Center Experience

**DOI:** 10.7759/cureus.41114

**Published:** 2023-06-28

**Authors:** Saji Vargheese, Thirugnanam Nelson, Anis Akhtarkhavari, Satya R Patra, Shivakumar M Algud

**Affiliations:** 1 General Surgery, Andaman & Nicobar Islands Institute of Medical Sciences, Govind Ballabh Pant Hospital, Port Blair, IND; 2 Surgical Disciplines, All India Institute of Medical Sciences, New Delhi, IND

**Keywords:** acute calculous cholecystitis, gall stone, gallbladder, laparoscopic cholecystectomy, acute cholecystitis

## Abstract

Background

Laparoscopic cholecystectomy (LC) has increasingly been accepted as the procedure of choice for the treatment of acute cholecystitis (AC). However, the timing of this procedure in the management of AC remains controversial. Hence this study was conducted to assess the feasibility of early laparoscopic cholecystectomy in acute cholecystitis.

Materials and methods

Patients who presented with symptoms of acute cholecystitis such as pain and tenderness in the right upper quadrant, systemic signs of inflammation, and positive ultrasound findings according to Tokyo guidelines were included for evaluation. Group 1 includes patients presented within 24 hours of the onset of symptoms whereas those presented between 25 and 72 hours of the onset of symptoms belonged to Group 2. All patients were taken up for early LC after assessment. Intraoperative and postoperative complications were analysed.

Results

Out of 120 patients, 37 belonged to Group 1 (30.83%) and 83 belonged to Group 2 (69.17%). There was a significant difference between the study groups in terms of certain demographic, laboratory findings and duration of surgery. None of the patients in Group 1 developed postoperative complications, whereas one patient in Group 2 had a bile leak on postoperative Day 2. Group 2 had a higher conversion rate to open procedure (p = 0.059). The mean duration of hospital stay for patients in Groups 1 and 2 were 3 and 3.3 days, respectively.

Conclusion

Laparoscopic cholecystectomy is safe and feasible with minimal conversion rates in patients presenting with early symptoms of AC. With the availability of good visualisation, optics, instruments and energy sources, good outcomes can be achieved.

## Introduction

Acute cholecystitis (AC) is commonly encountered in surgical practice with a clear indication for surgery [1]. Laparoscopic cholecystectomy (LC) has increasingly been accepted as the procedure of choice for the treatment of symptomatic gallstones and acute as well as chronic cholecystitis [2,3]. However, its role and timing in the management of AC remain controversial [4]. The potential hazard of complications due to obscured anatomy caused by acute inflammation is an important issue [5,6]. Performing this procedure during the acute inflammatory phase is associated with an increased incidence of conversion to open surgery, even in experienced hands [6-9]. This conversion eventually results in the deprivation of all the potential advantages of a minimally invasive procedure. Theoretically, the acute phase of AC has been conservatively managed, followed by interval cholecystectomy, when inflammation and edema have subsided [4].

Unequivocal evidence supports the superiority of early LC within 72 hours over delayed LC owing to the outcome and cost of treatment [10-14]. This trend has been reported to be successful in a recent study in patients managed within 24 hours of admission [15]. However, cholecystectomy may not always be possible within 24 hours of admission for many different reasons. In such cases, the option of performing surgery within 72 hours has been recommended in several guidelines [3,12,16]. Hence this study was conducted to assess the feasibility of early laparoscopic cholecystectomy in acute cholecystitis.

## Materials and methods

Objectives

The objectives of this study were to (1) assess the feasibility of early LC in AC, and (2) compare the efficacy of LC in patients with AC presenting at 24 hours and between 25-72 hours.

Patients and methods

This prospective study was conducted from July 2017 to June 2020 in a secondary care hospital in Port Blair, India. Patients who presented with symptoms of AC such as pain and tenderness in the right hypochondrium, systemic signs of inflammation and positive ultrasonic findings such as cholelithiasis, distended gallbladder with increased wall thickness > 4mm, and pericholecystic fluid collection were included in the study [17]. Group 1 includes patients who presented within 24 hours of the onset of symptoms whereas those who presented to the hospital between 25 and 72 hours of the onset of symptoms belong to Group 2. In total, 120 patients who presented to the hospital with the above features were evaluated, assessed and advised for early laparoscopic cholecystectomy. The majority of patients presented late to the hospital due to logistic reasons. With appropriate consent, patients were worked up for surgery. Standard laparoscopic cholecystectomy was performed using a 3D high-definition camera system. All surgeries were performed by a highly experienced laparoscopic surgeon. The energy sources used were ultrasonic scissors and bipolar cautery. Intraoperative and postoperative complications were analysed.

Operative Procedure

**Figure 1:**
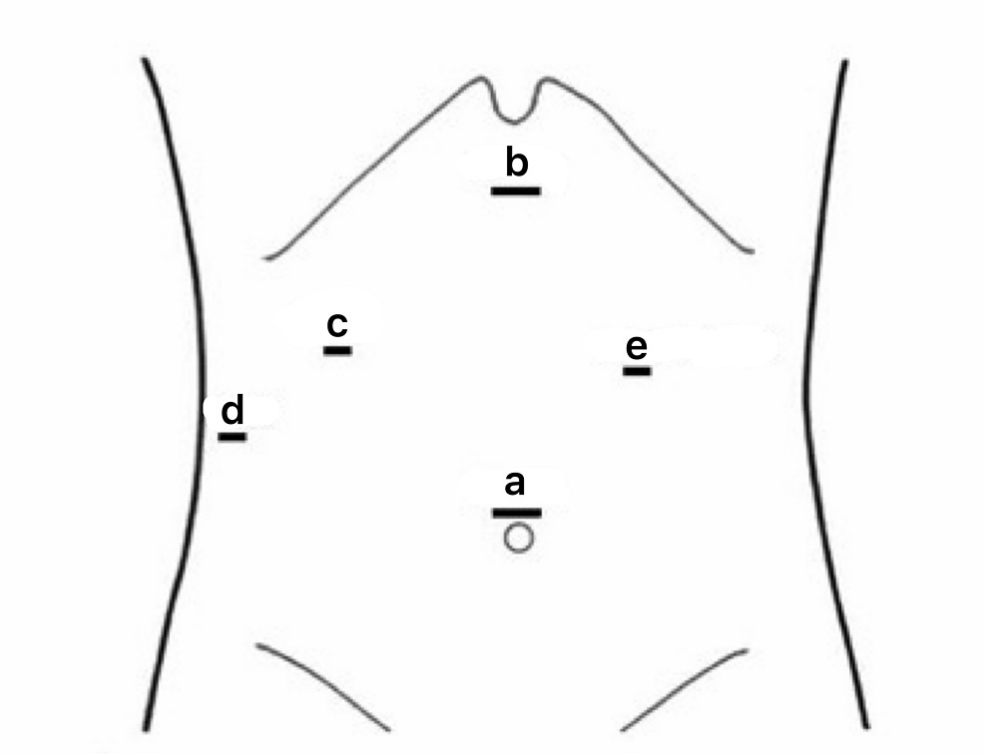
Port placements during LC in patients with acute cholecystitis a: 10 mm - Camera port at the supraumbilical region b: 10 mm - Right-hand operating port c: 5 mm - Left-hand operating port d: 5 mm - Port for retraction of fundus e: 5mm - Para rectus in the left hypochondrium for duodenum retraction (optional) LC - laparoscopic cholecystectomy Image credit: Saji Vargheese

Ports were made as shown in Figure 1.

Pneumoperitoneum was created using the Veress needle. The fundus of the gallbladder was retracted for dissection of Calot's triangle with a critical view of safety. The cystic duct and artery were ligated and cut using titanium and Hem-o-Lok clips. Gallbladder was dissected from the liver bed and retrieved using an endobag. Thorough saline lavage was given. Hemostasis was maintained throughout the procedure. Ports were closed after desufflation. The number of ports varied depending on the ease of approach and dissection. The gallbladder was decompressed by puncture and aspiration due to enormous distension or thickened wall in certain cases. A sub-hepatic drain was placed if profuse oozing was encountered.

For all patients, the duration of surgery and the number of ports were recorded. The gallbladder was assessed for distension, thickening, adhesions, and anatomical distortion using the severity grading of Tokyo guidelines [17]. Intraoperative events such as gallbladder decompression, ease of dissection, and placement of the subhepatic drain were observed and recorded. Postoperatively, patients were shifted to ICU for observation and then transferred to wards. During the postoperative period, all patients were observed for complications such as persistent fever, abdominal distension, bleeding, and bile leak. The observed results were tabulated and the two groups were evaluated to assess the feasibility of early LC in acute cholecystitis.

## Results

Among 120 patients, 37 belonged to Group 1 (30.83%) and 83 belonged to Group 2 (69.17%). Out of 120 patients, 50 were males (41.67%) and 70 were females (58.33%). Group 1 includes 16 males (43.24%) and 21 females (56.76%), whereas Group 2 includes 34 males (40.96%) and 49 females (59.04%) (Table 1). The mean age of patients in Group 1 was 31 years and Group 2 was 56 years. The mean duration of surgery in Group 1 was 19 minutes and 42 seconds whereas in Group 2, it was 36 minutes and 36 seconds. Table 2 demonstrates the comparison of physical and investigative findings between both groups. In Group 1, all patients were operated using standard 4 ports, whereas, 11 patients in Group 2 required an extra fifth port with a significant statistical difference (p=0.004). Decompression of the gallbladder was not done in Group 1 whereas 8 patients in Group 2 required decompression (p=0.020). Sub-hepatic drain placement was not required in Group 1, whereas 21 patients in Group 2 underwent drain placement during surgery with a statistically significant difference (p<0.001). The mean duration of hospital stay for patients in Groups 1 and 2 was 3 and 3.3 days respectively. Conversion to open procedure was higher in Group 2 whereas all patients in Group 1 underwent the procedure laparoscopically (p=0.059). None of the patients in Group 1 suffered postoperative complications, whereas 1 patient in Group 2 suffered a bile leak on the postoperative Day 2, following which the patient was subjected to ERCP and CBD stenting. There was no statistically significant difference between the two groups in terms of complications (p=1.000) (Table 3).

**Table 1:** Comparison of the basic clinical and demographic data of patients Data are presented as mean± SD unless indicated otherwise

Parameters	Group 1; N=37(%)	Group 2; N=83(%)	P-value
Age (years)			
11-30	19	3	<0.001
31-50	18	29	0.109
>50	0	51	<0.001
Male	16	34	0.815
Female	21	49	
Pain duration in hours	9.89 ± 4.08	34.89 ± 23.89	<0.0001
Previous attack	16 (43)	18 (21.6)	0.015
Previous abdominal surgery	6 (16)	5 (6)	0.073
Comorbidities	6 (16)	8 (9)	0.299

**Table 2:** Comparison of physical, laboratory and radiological findings of patients Data are presented as mean± SD unless indicated otherwise

Parameters	Group 1; N=37(%)	Group 2; N= 83(%)	P-value
Physical findings			
Tenderness	37 (100)	83 (100)	1.000
Rebound tenderness	18 (50)	40 (49)	0.963
Laboratory findings			
White blood cells (10^9^/L)	11.93 ± 4.8	13.43 ± 3.88	0.072
Total bilirubin (mg/dl)	0.99 ± 0.43	3.43 ± 11.22	0.189
Direct bilirubin (mg/dl)	0.41 ± 0.22	0.23 ± 0.12	0.922
Gamma glutamyl transferase (U/L)	120.3 ± 134.6	134.56 ± 111.2	0.545
Radiological findings			
Calculi in gallbladder	37 (100)	83 (100)	1.000
Gallbladder wall thickening	8 (21)	21 (25)	0.663

**Table 3:** Comparison of intraoperative and postoperative findings of patients p<0.05 shows the statistically significant difference

Parameters	Group 1; N=37(%)	Group 2; N=83(%)	P-value
Extra number of ports	0	11	0.004
Decompression of GB	0	8	0.020
Placement of subhepatic drain	0	21	<0.001
Conversion to open procedure	0	6	0.059
Mean duration of hospital stay (days)	3	3.3	1.000
Postoperative complications (bile leak, bleeding)	0	1	1.000

## Discussion

Management of acute cholecystitis with conservative treatment followed by delayed-interval laparoscopic cholecystectomy had been the common practice in the early 1990s. Early LC showed a higher complication rate, increased operative time and a higher rate of conversion in AC. With increasing surgical experience and the availability of energy sources, early LC is considered safe for the management of AC in the current decade [4].

For quite some time, AC was considered a contraindication to laparoscopic cholecystectomy. With improvements in instruments and techniques, the number of reports on LC for acute cholecystitis has increased, with conversion rates ranging from 6.5% to 35% [4,16,18]. In our study, the maximum incidence of AC was seen in the age group of 41-50 years, with the mean age of the study population being 48 years. Similar studies have shown increased incidence in the age group of 61-70 years [1,4]. Our study population showed an increased incidence in females (58.33%) than in males (41.67%) whereas many other studies have shown an increased incidence of AC in males [1,4,7].

The mean duration of surgery in Group 1 was 19 minutes and 42 seconds whereas in Group 2, it was 36 minutes and 36 seconds. This is due to the severity of inflammation in due course of time having its impact on dissection, identification of the Calot's triangle and bleeding [1,4]. Similar explanations can be given for the extra number of ports, decompression of the gallbladder and placement of a subhepatic drain in Group 2. The length of hospital stay in Group 1 was 3 days and in Group 2, it was 3.3 days. Previous studies demonstrated the shortened length of hospital stay in early LC patients [4]. None of the patients in Group 1 suffered postoperative complications, whereas one patient in Group 2 suffered a bile leak on postoperative Day 2 (1.2%), following which the patient was subjected to ERCP and stenting. Similarly, a previous study showed there was no significant difference in the rate of complications, operating time and conversion rate for early LC in acute cholecystitis [4]. 

These results emphasise the fact that early LC is the feasible treatment for acute cholecystitis, provided it is done in the first 24 hours of inflammation, which has been referred to as the ‘golden period’. LC done between 25 and 72 hours is also proven to be beneficial (known as the ‘silver period'), but the outcomes are comparatively poorer.

In our study groups, we had an acceptable conversion rate of 5%. This can be attributed to the optics, visualisation, prompt energy sources and mainly the experience and skill of the surgeon performing the surgery. Previous studies emphasise that the surgeon should know the threshold for conversion based on intraoperative findings and must be ready to convert at any time, keeping in mind the benefit of the patient and a successful outcome [13,16]. But ultimately, in the hands of experienced surgeons, LC is now feasible in a majority of cases in AC. LC can be done with utmost safety and precision, both in the golden and silver periods, as well as in the case of delayed presentation of cholecystitis.

Limitations

In our study, the majority of the patients presented late, more than 24 hours after the onset of symptoms to the hospital. Further, a randomised controlled trial in a high-volume centre is needed to study the efficacy of LC in the case of early and delayed presentations of patients with AC. 

## Conclusions

Laparoscopic cholecystectomy is safe and feasible with minimal conversion rates in patients presenting with early symptoms of AC. The present study supports performing laparoscopic cholecystectomy early in the course of AC, preferably within 24 hours. With the availability of good visualisation, optics, instruments and energy sources, good outcomes can be achieved.
